# RNA structure refinement using NMR solvent accessibility data

**DOI:** 10.1038/s41598-017-05821-z

**Published:** 2017-07-14

**Authors:** Christoph Hartlmüller, Johannes C. Günther, Antje C. Wolter, Jens Wöhnert, Michael Sattler, Tobias Madl

**Affiliations:** 1 Center for Integrated Protein Science Munich, Department Chemie, Technical University of Munich, Lichtenbergstr. 4, 85748 Garching, Germany; 2 Institute of Structural Biology, Helmholtz Zentrum München, Ingolstadter Landstr. 1, 85764 Neuherberg, Germany; 3 Institut für Molekulare Biowissenschaften and Zentrum für Biomolekulare Magnetische Resonanz (BMRZ), Goethe-Universität Frankfurt, Max-von-Laue Str. 9, 60438 Frankfurt/M, Germany; 4 Institute of Molecular Biology and Biochemistry, Center of Molecular Medicine, Medical University of Graz, Harrachgasse 21, 8010 Graz, Austria

## Abstract

NMR spectroscopy is a powerful technique to study ribonucleic acids (RNAs) which are key players in a plethora of cellular processes. Although the NMR toolbox for structural studies of RNAs expanded during the last decades, they often remain challenging. Here, we show that solvent paramagnetic relaxation enhancements (sPRE) induced by the soluble, paramagnetic compound Gd(DTPA-BMA) provide a quantitative measure for RNA solvent accessibility and encode distance-to-surface information that correlates well with RNA structure and improves accuracy and convergence of RNA structure determination. Moreover, we show that sPRE data can be easily obtained for RNAs with any isotope labeling scheme and is advantageous regarding sample preparation, stability and recovery. sPRE data show a large dynamic range and reflect the global fold of the RNA suggesting that they are well suited to identify interaction surfaces, to score structural models and as restraints in RNA structure determination.

## Introduction

In the last decades, ribonucleic acids (RNAs) have been found to be key regulators for numerous important cellular processes including transcription, translation, splicing, cell differentiation as well as cell death and proliferation^1^. Besides regulatory functions, RNAs are essential for catalysis in ribosomes and spliceosomes, sensing environmental parameters such as temperature or metabolite concentration, as well as storing the genomic information of RNA viruses. Understanding the underlying molecular mechanisms of these processes requires insights into the structure and dynamics at an atomic level. In the last decades, NMR spectroscopy has developed to a powerful technique to study RNA structure and dynamics^2–8^. However, technical challenges are caused by poor chemical shift dispersion leading to spectral overlap as well as by the low proton density and the low number of intramolecular interactions limiting the number of observable distance restraints that can be used to define RNA structure. Moreover, the RNA backbone is defined by several torsion angles, requiring a large set of restraints to obtain an accurate structural model. To overcome these limitations, different labeling strategies, including for example specific or uniformly ^13^C- and/or ^15^N-labeling and segmental labelling of RNAs were developed to reduce spectral complexity and signal overlap^9–22^. Large sets of restraints including NOE-based distances, torsion angles and orientational restraints derived from RDCs and J-couplings, respectively, as well as base pairing contacts obtained from J-couplings across hydrogen bonds are typically collected to provide a sufficient number of restraints for high-resolution models of RNAs^2, 6, 23–26^.

**Figure 1:**
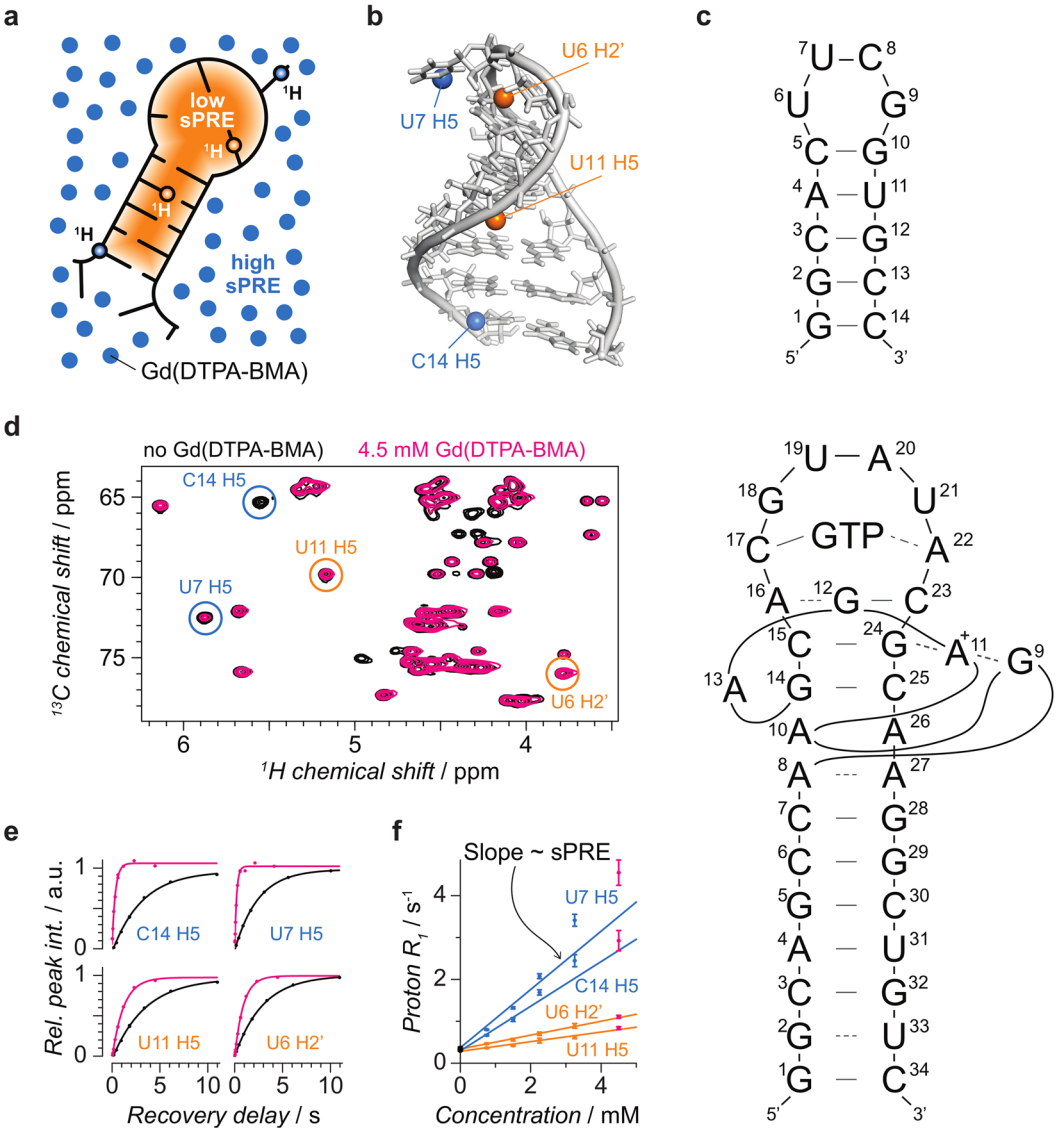
Concept of sPRE and exemplary sPRE data of the UUCG tetraloop. (**a**) sPRE data provide a quantitative measure for solvent accessibility and encode distance-to-surface information. sPRE data are acquired by titrating the RNA with the paramagnetic compound Gd(DTPA-BMA). (**b**) The NMR solution structure of the UUCG tetraloop (PDB code 2KOC) is shown and for illustration purpose, two solvent-exposed protons (blue spheres) and two buried protons (orange spheres) are highlighted. (**c**) Schematic representation of the UUCG tetraloop (top) and the GTP-bound GTP aptamer. (**d**) NMR spectra of the UUCG tetraloop are shown in the absence (black) and presence (magenta) of Gd(DTPA-BMA) with circles around the peaks corresponding to the protons shown in (**b**). (**e**) Quantitative sPRE data are obtained by measuring the longitudinal proton *R*_1_ relaxation rate as a function of the concentration of Gd(DTPA-BMA). (**f**) Proton *R*_1_ rates increase linearly with the concentration of the paramagnetic compound and the slopes correspond to the sPRE values of the respective resonances highlighted in (**b**).

In previous NMR studies, solvent accessibility data were obtained by measuring the NOE between water and protein protons, but the data were found to be dominated by chemical exchange^27, 28^. Here, we demonstrate that solvent accessibility data derived from solvent paramagnetic relaxation enhancements (sPREs) provide valuable information to characterize the conformation of RNAs and will be useful to enable NMR studies of larger RNAs. sPRE data are a quantitative measure for solvent accessibility and thus readily provide distance-to-surface information^29^ (Fig. 1). Directly observing the solvent accessible areas is a promising approach not only for mapping interaction surfaces but also for structure determination, as has been shown for proteins^30–32^. Recently, computational protocols using sPRE data were developed for XplorNIH^27^ and the Rosetta framework^33^. The implementation and use of sPRE data has several advantages: i) no covalent labeling of RNAs is required, ii) the sample can be recovered, iii) complete chemical shift assignment is not required and iv) any type of NMR spectrum such as proton 1D, ^1^H, ^15^N HSQC or ^1^H, ^13^C HSQC experiments can be used. Direct measurements of solvent accessibility data of RNAs have been reported previously using the soluble compound TEMPOL^34^. While the correlation of TEMPOL-induced sPREs agreed with RNA structure for some parts, the data were limited to a qualitative validation of models. Moreover, unexpectedly large sPREs were found for certain nucleotides and it was later suggested that TEMPOL forms intermolecular hydrogen bonds leading to preferred binding to specific sites^35^. Here, sPRE data are obtained by titrating the RNA sample with Gd(DTPA-BMA), a soluble, well-characterized Gd^3+^ chelating paramagnetic contrast agent that was originally developed for MRI imaging^31, 36^.

## Results

### Gd(DTPA-BMA) provides quantitative solvent-accessibility data of RNAs

To first assess a potential binding to specific RNA moieties, fingerprint spectra in the absence and presence of the paramagnetic compound were recorded for two RNAs exhibiting distinct folds and structural elements. The resulting NMR spectra of the UUCG tetraloop^37, 38^ and the GTP class II aptamer^39, 40^ are shown in Supplementary Figures 1 and 2, respectively. As expected, the presence of Gd(DTPA-BMA) causes line-broadening of NMR signals. However, even in the presence of the paramagnetic compound, the vast majority of peaks are still well observable and the absence of chemical shift perturbations confirms the absence of specific binding of the compound to the RNA (Fig. 1d and Supplementary Figures 1 and 2). Thus Gd(DTPA-BMA) can be used as a paramagnetic probe that screens the accessible surface area of RNA molecules.

As unchelated lanthanide ions can efficiently hydrolyze RNA^41^, we checked for potential cleavage products of the RNAs in the presence of the Gd^3+^ compound. No degradation products were observed for any RNA studied and sPRE data obtained from a single RNA sample were reproducible even after several sPRE measurements, each followed by a removal of Gd(DTPA-BMA) (data not shown). The absence of phosphodiester hydrolysis can be explained by the very high chelating stability of DTPA-BMA^36^ and the presence of a slight excess of Ca^2+^-bound chelator.

The sPRE data were acquired by measuring proton longitudinal relaxation rates (^1^H-*R*_1_) as a function of increasing concentrations of Gd(DTPA-BMA). As expected, ^1^H-*R*_1_ rates were found to increase linearly with the concentration of Gd(DTPA-BMA) in the case of carbon-bound protons. For imino and amino protons, the increase of ^1^H-*R*_1_ rates is larger at small concentrations of the paramagnetic compound (below about 1 mM), and becomes linear at higher concentrations (Supplementary Figure 3). This observation likely reflects an additional exchange contribution that mixes the relaxation mechanisms of water and RNA protons. Nevertheless, a quantitative measure for the solvent accessibility can be obtained for nitrogen-bound exchangeable protons by determining the minimum concentration of Gd(DTPA-BMA) for which a linear response is observed. Only titration points at this concentration or above are used to fit the linear sPRE model (0.5 mM or above for the UUCG tetraloop and 1.29 mM or above for the GTP aptamer; compare Supplementary Tables 1 and 2). For details about data acquisition and analysis, refer to the methods section in the supplementary information.

### sPRE data correlate well with RNA structure

**Figure 2:**
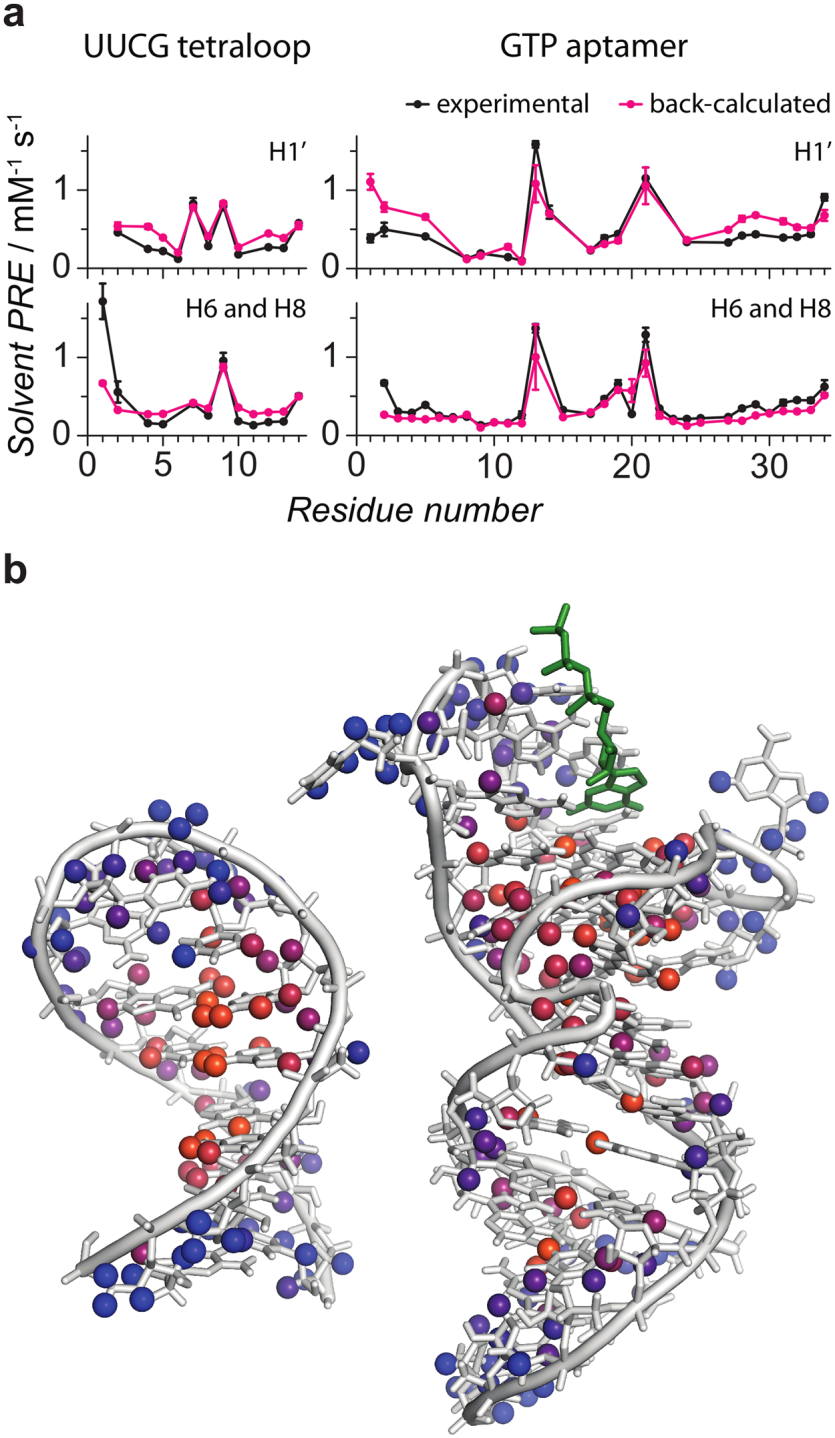
sPRE data correlates well with RNA structure. (**a**) Experimental sPRE data (black) are compared to predicted sPRE data based on NMR solution structures of the UUCG tetraloop (2KOC) and the GTP-bound aptamer (5LWJ). Data are shown for sugar protons (H1′) and aromatic protons (H6 and H8) as indicated. (**b**) NMR solution structures of the UUCG tetraloop^37, 38^ (2KOC, left) and the GTP-bound aptamer^39, 40^ (5LWJ, right, heavy atoms of GTP ligand shown in green) are shown. All protons for which a sPRE value was obtained are shown as spheres and colored according to the sPRE (blue corresponding to high and orange corresponding to low sPRE values).

Next, we analyzed how sPRE data reflect the structural features of the RNAs studied. To this end, the NMR structures of the UUCG tetraloop and the GTP-bound GTP class II aptamer were used to predict the expected sPRE data using a previously published grid-based approach^30, 31^ (see method section for details). The experimental sPRE data correlate very well with the predicted data (Fig. 2a and Supplementary Figures 4a and 5). This demonstrates that sPRE data provide a quantitative measure of solvent-accessibility that correlates well with RNA structure. In particular, all buried protons of both RNAs show a small sPRE. Protons for which significant deviations between experimental and back-calculated sPREs are observed are found in loop regions or in terminal nucleotides. These regions are flexible and thus are expected to show a population-weighted average of the sPRE. The structural NMR ensembles used to predict the sPRE data represent the most stable conformations in solution and depend on the algorithm and experimental restraints used for the structure determination. Thus, the NMR ensembles do not necessarily reflect the equilibrium distribution of the different conformations of these solvent-exposed regions of the RNA. Since all conformations of the ensemble were equally weighted in the prediction of the sPRE data, the prediction is expected to deviate for dynamic regions of the RNA. It is further worth noting, that despite the compact fold of the UUCG tetraloop, the experimental sPRE data show a large dynamic range, which renders them a powerful source to characterize the structure of RNAs. The excellent correlation between experimental data and RNA structure in combination with a large dynamic range suggests sPRE data as promising parameters to probe RNA structures by NMR.

As described above, significant deviations between predicted and measured sPRE data are observed for some terminal nucleotides. Among all 258 observed sPRE values (93 for the UUCG tetraloop and 165 for the GTP aptamer), only two protons that are located in non-terminal nucleotides show significant deviations (9Gua H1 and 6Ura H3 in the UUCG tetraloop). Both protons are located in the loop region of the RNA in close spatial proximity to each other (Supplementary Figure 4b) and the corresponding sPRE values are underestimated based on the NMR solution structure, independent of which structural model of the UUCG loop motif (PDB codes 1HLX, 1K2G, 1TLR, 1Z31, 2KHY, 2KOC, 2KZL, 2LHP, 2LUB and 2N6X) was used. Although the sPRE of these protons are expected to have an additional contribution due to chemical exchange with water, the sPRE values of 2.2 and 0.44 s^−1^mM^−1^ of 6Ura H3 and 9Gua H1, respectively, are significantly larger compared to the average of 0.079 ± 0.037 s^−1^mM^−1^ for all other imino protons. This suggests that conformational dynamics in this regions may cause the unusually high experimental sPREs. Indeed, recent molecular dynamics studies that included a large set of NMR relaxation data of the UUCG tetraloop, suggest the loop region to be more flexible than the RNA stem^42–48^. Moreover, intermediate structural states with the bases of the loop region experiencing higher solvent exposure were observed in these studies. This suggests that the increased sPREs are a result of increased flexibility and dynamics of the loop region. However, future studies are required to fully understand the usability of sPRE data to detect dynamics in RNAs.

### sPRE data provide orthogonal restraints for RNA structure determination

**Figure 3:**
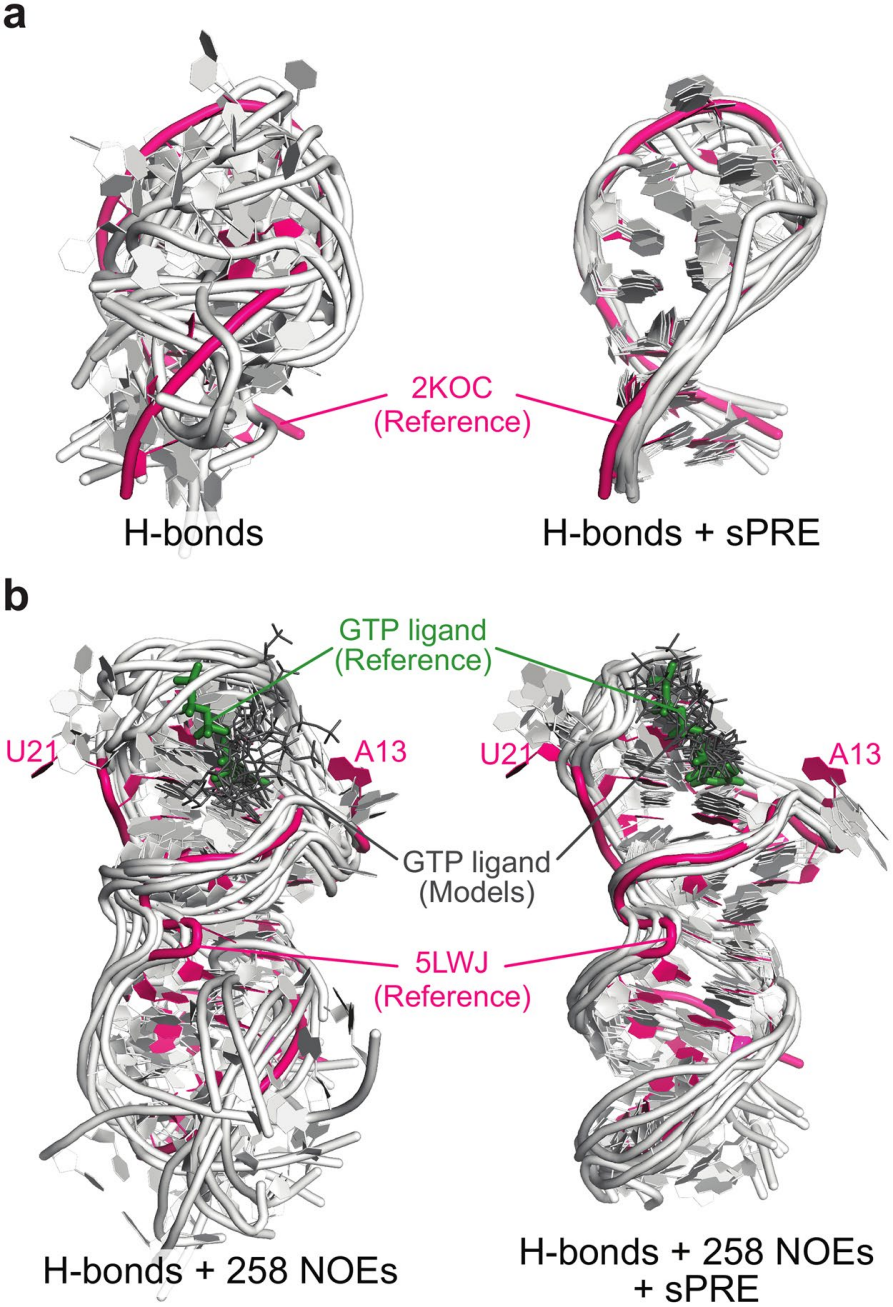
sPRE data improve structure determination of RNAs. Structural models of the UUCG tetraloop (**a**) and the GTP-bound GTP aptamer (**b**) were obtained without (left) and with (right) sPRE data using XplorNIH. The 10 best scored models (light gray) in terms of total energy were selected from a total of 200 models and aligned to the corresponding NMR structure (magenta). All restraints used in the computations are indicated below the respective models. In (**b**) heavy atoms of the GTP ligand are shown as sticks (Reference in green, computed models in dark gray) and the positions of the intrinsically flexible nucleotides A13 and U21^40^ are indicated.

Next, the potential of sPRE data to facilitate the structure determination of RNAs was investigated (Fig. 3). To this end, structural models of the UUCG tetraloop as well as the GTP-bound aptamer were computed using the XplorNIH framework^27, 49–51^ in combination with different sets of experimental NOE data (Supplementary Tables 3 and 4, Supplementary Figure 7). The results show that sPRE data significantly improves convergence and accuracy of the structure determination, in particular in cases where only limited sets of NOEs are available. For example, using only 258 experimental NOEs of the GTP-bound aptamer (30% of the all NOEs), the average RMSD of the obtained models was significantly reduced from 7.80 Å to 3.51 Å (Fig. 3b; Random set 2 in Supplementary Table 3). Next, the performance of the sPRE potential in combination with other experimental NMR-based restraints was investigated (Supplementary Table 5). The benchmark revealed that solvent accessibility data is an orthogonal restraint since its usage improves structure determination of the UUCG tetraloop in combination with NOE, RDC and torsion angle restraints. Strikingly, using only hydrogen bonds-derived restraints in combination with sPRE data, the structure of the UUCG tetraloop can be determined with an RMSD of 2.8 Å compared to the published NMR-based structure (Fig. 3a). Even sparse sPRE data sets, in some cases down to 25%, improve structural quality of the UUCG tetraloop (Supplementary Table 6).

## Discussion

In summary, we show that sPRE data are efficient NMR observables to probe RNA structure. sPRE data obtained with the paramagnetic compound Gd(DTPA-BMA) yield quantitative information of solvent accessibility as they correlate well with RNA structure, provide structural information for both buried and surface-exposed atoms and can be used as restraints to drive molecular dynamics-based structure determination of RNAs. Since sPRE data reflect the global fold of a RNA they are well suited to identify tertiary contacts or map interaction surfaces with other molecules^32^, for example, in RNA-protein complexes. As the measurement of sPRE data does not require complete chemical shift assignments, surface accessibility restraints can be recorded for structural studies also in the case of large RNAs, in particular in combination with specific or segmental labelling, where it is difficult to obtain a comprehensive set of experimental restraints for structural analysis. Compared to proteins, several considerations have to be taken into account for sPRE-based studies of RNAs. First, different NMR pulse sequences might be needed, in particular for triple resonance experiments. Second, multiple data sets acquired for different RNA labelling schemes and different solvent conditions need to be combined. In order to compensate for minor differences between these data sets we have proposed an approach in which we normalize the sPRE data using the sPRE of water protons in each sample. Third, lower concentrations of Gd(DTPA-BMA) are used for RNAs compared to proteins due to the higher sPRE caused by the lack of large hydrophobic cores resulting in smaller distances to the RNA surface. Last, chemical exchange of protons with water is typically more efficient in RNAs and modulates sPREs of nitrogen-bound amino and imino protons. In these cases, only the region of linear response is used for quantitative analysis.

Our results show that sPRE-derived restraints are particularly promising to define the global fold of larger RNAs and provide valuable information for structure determination that is orthogonal to other NMR-based restraints in molecular dynamics-based approaches. Recently, accuracy of chemical-shift based RNA *de novo* structure prediction using the Rosetta framework was significantly enhanced by improving the chemical shift scoring function for RNAs^52^. Combining this improved score with a sampling algorithm driven by sPRE data^33^ and specific or segmental labeling could provide powerful tools for structural modeling of large RNAs based on NMR data in the future.

## Methods

### RNA sample preparation

A uniformly ^13^C, ^15^N labeled sample of the UUCG tetraloop (5′-GGCACUUCGGUGCC-3′) was purchased from Silantes (Munich, Germany). The NMR buffer contained 20 mM K_2_HPO_4_ and 0.4 mM EDTA and was adjusted to pH 6.4. Transcription and purification of the GTP class II aptamer in complex with GTP was been described previously^39^. The sample was measured in NMR buffer containing 25 mM KH_2_PO_4_, 25 mM KCl and 2 mM magnesium acetate. The buffer was adjusted to pH 6.3 and uniformly ^13^C, ^15^N labeled RNA was measured in the presence of a two-fold excess of unlabeled GTP. NMR assignments and a solution NMR structure of the UUCG tetraloop were obtained from literature^37, 38^ (PDB code 2KOC, BMRB 5705). For the GTP class II aptamer in complex with GTP, the recently reported NMR assignments and solution NMR structure were used^39, 40^ (PDB code 5LWJ, BMRB 25661).

### NMR Experiments

NMR sPRE data were obtained by measuring proton *R*_1_ relaxation rates as a function of the concentration of Gd(DTPA-BMA) using a saturation-recovery approach which has been described previously for protein applications^30^. Briefly, a 7.5 ms proton trim pulse followed by a gradient is used to dephase magnetization. Next, longitudinal magnetization is recovered during a recovery delay of variable length. The recovery delay is followed by a read-out spectrum, such as a ^1^H,^15^N or ^1^H,^13^C HSQC. This saturation-recovery scheme allows to use a short interscan delay (about 50 ms) which dramatically reduces experimental time compared to measuring proton R_2_ rates. Pseudo-3D experiments were acquired for 4 to 7 different concentrations of the paramagnetic compound. To recover the NMR RNA sample, the paramagnetic compound was first removed by dialysis against water using a membrane with a 1 kDa molecular weight cut-off and then, the sample was lyophilized and resuspended in the corresponding NMR buffer.

sPRE data for nitrogen-bound protons of the UUCG tetraloop were recorded with a 0.8 mM uniformly ^13^C, ^15^N labeled sample at 283 K on an Avance III Bruker 900 MHz NMR spectrometer equipped with a cryo-TXI probe head. Proton *R*_1_ relaxation rates were measured in the absence and presence of 0.5, 1, 1.75, 2.75 and 4.0 mM Gd(DTPA-BMA) using ^1^H,^15^N HSQC-based pseudo-3D experiments. sPRE data for carbon-bound protons were acquired with the same sample at 298 K and *R*_1_ relaxation rates were measured in the absence and presence of 0.75, 1.5, 2.25, 3.25 and 4.5 mM Gd(DTPA-BMA) using ^1^H,^13^C HSQC-based pseudo-3D experiments. Delay times and other NMR parameters for all experiments as well as the total NMR time are presented in Supplementary Table 1. To obtain sPRE data of the GTP aptamer in complex with GTP, samples containing about 150 µM of (^13^C, ^15^N)-GU, (^13^C, ^15^N)-C or (^13^C, ^15^N)-A labeled RNA and 300 µM unlabeled GTP were measured at 293 K on an Avance III Bruker 900 MHz NMR spectrometer equipped with a cryo-TXI probe head and an Oxford Instruments 600 MHz NMR spectrometer equipped with a Bruker Avance III console and a cryo-TCI probe head. ^1^H,^13^C and ^1^H,^15^N HSQC-based pseudo-3D experiments were used to acquire proton *R*_1_ relaxation rates for 4 to 7 different concentrations of Gd(DTPA-BMA). Delay times and other NMR parameters for all experiments as well as the total NMR time are presented in Supplementary Table 2. For all RNA samples, amino and imino protons were measured in buffer containing 5 to 10% D_2_O whereas carbon-bound protons were acquired in buffer containing ≥99.990% D_2_O (Sigma-Aldrich). For every titration step of Gd(DTPA-BMA), solvent water *R*_1_ was measured and the derived sPRE of the water solvent was used to normalize the RNA sPRE values. The normalized sPRE data were then rescaled by the mean sPRE of the solvent, obtained by averaging the sPRE of water protons in all relevant experiments. This procedure allows to combine sPRE measurements of different samples or different magnetic field strengths (e.g. in different buffers or with different labeling schemes; see Supplementary Figure 6). Furthermore, by plotting the solvent water *R*_1_ against the concentration of Gd(DTPA-BMA) errors in pipetting can be detected and compensated accordingly if necessary.

In the case of nitrogen-bound protons, plotting the proton *R*_1_ against the concentration of Gd(DTPA-BMA) revealed a non-linear correlation with rates at low concentrations of the paramagnetic compound being lower than expected according to a linear model. Consequently, the *R*_1_ for low concentrations (0 mM in the case of the UUCG tetraloop, as well as 0 and 0.645 mM in the case of the GTP aptamer) were omitted in the computations of the sPRE values and only the linear correlation was used (compare Supplementary Table 1 and 2 as well as Supplementary Figure 3). In all cases, at least 4 different concentrations were used to derive the sPRE.

For large RNAs, specific isotope labeling schemes are required to reduce signal overlap^9, 10, 53^. In a simple and straight-forward approach, only one or two nucleotide types are enriched in NMR active nuclei. While this approach reduces spectral complexity, the measurement of sPREs with different samples might introduce systematic errors, due to pipetting errors. In addition, the usage of different magnetic field strengths affects the scaling of the sPRE data^31^. To ensure that sPRE data acquired on multiple samples and field strengths are consistent, the sPRE data sets are scaled by referencing to the sPRE of the water solvent in each sample. This referencing notably improves the correlation of calculated and experimental sPRE (Supplementary Figure 6). To normalize the sPRE data, the sPRE of water protons was acquired by measuring the *R*_1_ rate of the water proton signal. The *R*_1_ rate was measured using a pseudo 2D experiments with simple 1D proton read-out (the proton pulse length was set between 0.3 and 1 µs). The change of the *R*_1_ rate of the water protons during the titration Gd(DTPA-BMA) with was used to obtain the sPRE of the water protons. The sPRE of the water solvent was then used to normalize the sPRE values of the RNA signals. The normalized sPRE values of different titration experiments (with different labeling schemes or at different field strengths) were then combined to generate a large sPRE data set for the corresponding RNA. To obtain an absolute quantity, the normalized sPRE data were then multiplied by the mean sPRE of the solvent, obtained by averaging the sPRE of water protons in all relevant experiments. This procedure allows to combine sPRE measurements of different samples or different magnetic field strengths (e.g. in different buffers or with different labeling schemes; see Supplementary Figure 6). Furthermore, by plotting the solvent water *R*_1_ against the concentration of Gd(DTPA-BMA) errors in pipetting can be detected and compensated accordingly if necessary.

The measurement times for the collection of sPRE data can be quite long, as sPRE data are recorded as a series of proton *R*_1_ relaxation experiments. Typical ^1^H-*R*_1_ rates in the absence of Gd(DTPA-BMA) are relatively slow with an average ^1^H-*R*_1_ rate of 0.23 s^−1^ (±0.10 s^−1^) at 900 MHz and 0.30 s^−1^ (±0.14 s^−1^) at 600 MHz. As a consequence, long delay times of several seconds (Supplementary Table 2) are required which in turn increase the overall experimental time. To overcome this drawback, the data sets of both RNAs were re-evaluated by determining the sPREs based on *R*_1_ rates measured only in the presence of 0.75, 1.5, 3.25 and 4.5 mM Gd(DTPA-BMA) for the UUCG tetraloop and 0.8, 1.6, 3.2 and 4 mM for the GTP aptamer. Notably, the obtained sPRE data are fully consistent with those derived from a full set of measurements, but the optimized acquisition scheme reduces experimental time by about 40%. It should be noted, that experimental time could be further reduced by applying selective saturation schemes that employ longitudinal relaxation-enhancing techniques as demonstrated in a previous study^54^.

### NMR data analysis

NMR spectra were processed with the NMRpipe software package^55^. Peak positions from literature were transferred using CcpNmr Analysis^56^ and peak intensities of well-resolved peaks were obtained using the nmrglue Python package^57^. Peak intensities of the pseudo-3D experiments were fitted to an exponential recovery function according to equation (1):

1 $$I(\tau )={I}_{0}-A\cdot {e}^{-{R}_{1}\cdot \tau }$$

where *τ* is the recovery delay, *I*(*τ*) is the peak intensity measured for the recovery delay *τ*, *I*_0_ is the maximum peak intensity, *R*_1_ is the longitudinal proton relaxation rate and *A* is the amplitude of the relaxation.

To estimate the error of the peak intensities *ε*, the recovery delay *τ*_*d*_ was measured twice to obtain two intensity values *I*(*τ*_*d*_, *I*, 1) and *I*(*τ*_*d*_, *I*, 2), where *i* is the index of the peak. For every peak *i*, the difference of the duplicates ${\rm{\Delta }}{I}_{i}=\,I({\tau }_{d},\,i,1)-I({\tau }_{d},i,\,2)$ was computed. The overall error of the pseudo-3D experiment *ε*_pseudo-3D_ was then computed according to equation (2):

2 $${\varepsilon }_{\mathrm{pseudo}-\mathrm{3D}}=\sqrt{\frac{1}{2N}\,\cdot \,\sum _{i}^{N}{({\rm{\Delta }}{I}_{i}-\overline{{\rm{\Delta }}I})}^{2}}$$

where *N* is the number of peaks, and $\overline{{\rm{\Delta }}I}=\sum _{i}^{N}{\rm{\Delta }}{I}_{i}$ is the average difference of the duplicates. The subtraction of $\overline{{\rm{\Delta }}I}$ accounts for systematic errors, and in all cases was significantly lower in magnitude than the differences of the duplicates ${\rm{\Delta }}{I}_{i}$.

The error of the peak intensities *ε*_pseudo-3D_ was then used to estimate the error of the fitted proton relaxation rates Δ*R*_1_. To this end, the experimental data was resampled using the following combined Monte Carlo-type bootstrapping approach: For every peak in a given pseudo-3D experiment, a set of delay-intensity data points, *I*(*τ*), was measured. This set is used to generate 1000 new random sets $\tilde{{I}_{j}(\tau )}$, each having 2.5 times as many data points as the original data set *I*(*τ*) (Recovery delays measured as duplicates are only counted as one). These sets were created by randomly selecting data points from the original data set *I*(*τ*) and allowing values to be selected multiple times (Increasing the size of the new sets by a factor of 2.5 allows to generate a more diverse ensemble of sets). For every set $\tilde{{I}_{j}(\tau )}$, the intensity values were randomly altered by adding a random number drawn from a normal distribution centered at 0 and with a standard deviation of *ε*_pseudo-3D_. Every set $\tilde{{I}_{j}(\tau )}$ was then fitted according to equation (1) giving rise to 1000 different ${R}_{1}^{j}$ rates. The error of the proton relaxation rate Δ*R*_1_ was computed as the standard deviation of all values ${R}_{1}^{j}$. This procedure was repeated for every peak and every pseudo-3D experiment.

To obtain the sPRE value, the proton *R*_1_ rates and the corresponding errors Δ*R*_1_ were collected for all measured concentrations of Gd(DTPA-BMA) *c*_para_ and a weighted linear regression using equation (3) was performed

3 $${R}_{1}({c}_{{\rm{para}}})={m}_{{\rm{sPRE}}}\cdot {c}_{{\rm{para}}}+{R}_{1}^{0}$$

where *R*_1_(*c*_para_) is the proton *R*_1_ measured at the concentration *c*_para_ of the paramagnetic compound, the slope *m*_sPRE_ corresponds to the sPRE and ${R}_{1}^{0}$ is the fitted proton *R*_1_ in the absence of the paramagnetic compound. The errors Δ*R*_1_ (obtained from the resampling approach described above) were used as weights in a weighted linear regression and the error of the sPRE value ${\rm{\Delta }}{m}_{{\rm{sPRE}}}$ as well as the error of the relaxation rate ${\rm{\Delta }}{R}_{1}^{0}$ were directly obtained from the weighted linear regression.

### Prediction of sPRE Data

To predict sPRE data based on published NMR solution structure, a previously published grid-based approach was used^31^. Briefly, the structural model was placed in a regularly-spaced grid representing the uniformly distributed paramagnetic compound and the grid was built with a point-to-point distance of 0.1 Å and a minimum distance of 20 Å between the RNA model and the outer border of the grid. Next, grid points that overlap with the RNA model were removed assuming a molecular radius of 3.5 Å for the paramagnetic compound. To compute the sPRE for a given RNA proton ${{\rm{sPRE}}}_{{\rm{predicted}}}^{i}$, the distance-dependent paramagnetic effect^29^ was numerically integrated over all remaining grid points according to equation (4):

4 $${{\rm{sPRE}}}_{{\rm{predicted}}}^{i}=c\,\cdot \sum _{{d}_{i,j}\, < \,20\,{\rm{\AA }}}\frac{1}{{{d}_{i,j}}^{6}}$$

where *i* is the index of a proton of the RNA, *j* is the index of the grid point, *d*_*i, j*_ is the distance between the *i*-th proton and the *j*-th grid point and *c* is an arbitrary constant to scale the sPRE values. Here, *c* was chosen such that the sum of all predicted sPRE values is equal to the sum of all experimental sPRE values.

### Structure Determination Benchmark

To demonstrate the potential of sPRE data for the structure determination of RNAs, structural models of the UUCG tetraloop as well as the GTP class II aptamer in complex with GTP were computed using the XplorNIH 2.40 framework^49, 50^. The used protocol is based on the gb1_rdc example that is included in the XplorNIH package and was adjusted to fold the respective RNA during an annealing procedure starting from an extended structure. The initial temperature was set to 3500 and the simulation at this high temperature was stopped after reaching 1000 ps or 10,000 steps. The temperature was then reduced to a final temperature of 25 in steps of 12.5 degrees and simulations were run for at least 4 ps or 2000 steps at every temperature step. The annealing procedure was repeated to obtain 200 models. The protocol made use of the recently published torsion potential RNA-ff1^51^ and the sPRE data was included in the structure determination using the nbTargetPot module^27^ of the framework. nbTargetPot is the scaling factor (or weight) of the sPRE potential. The value of the scaling factor mainly depends on the scaling factor of the other energy terms. The weight of the sPRE potential should not be proportional to the size of the RNA. For the mentioned benchmark, the weight of the sPRE potential was determined by testing different weights and the weight that produced the best models was used for all runs. To this end, one weight was determined for every RNA and the obtained weight was used for all computations of the corresponding RNA. The lower value of the UUCG tetraloop can be explained with the fact that the Xplor runs of the UUCG tetraloop included more energy functions than the ones of the GTP aptamer (in the case of the UUCG tetraloop, torsion angles and RDC, both obtained from PDB entry 2KOC, were used). The sPRE data sets of the UUCG tetraloop and GTP aptamer were filtered to remove all data points with an experimental error above 10% and all data points of nitrogen-bound protons. The nbTargetPot energy was initialized using the slope and intercept parameter as returned by the calibrate function of the nbTargetPotTools module in combination with the NMR structure of the corresponding RNA. The weight of the nbTargetPot energy was set to 1000 in the case of the UUCG tetraloop and 3000 for the GTP aptamer. For more details such as weight factors of all potentials, please refer to the python code of the protocols which are shown at the end of this document for both RNAs.

The annealing protocol was then used to benchmark the benefit of the sPRE data for structure prediction. To this end, structural models of the UUCG tetraloop and the GTP-bound aptamer were computed in the absence and presence of the sPRE potential and in combination with experimental restraints derived from hydrogen bond information and experimental NOEs (PDB entries 2KOC and 5LWJ)^37, 38, 40^. To simulate several NOE assignments, random experimental NOE subsets were created by randomly drawing a certain percentage of restraints from the full NOE set (compare Supplementary Tables 3 and 4). To account for the effect of the random selection process, at least 3 different random sets with the same number of NOE restrains were created and used to benchmark the impact of the sPRE data. It should be noted, that the randomly created NOE subsets not only account for different levels of assignments, but also simulate different qualities of NOE data as random subsets with the same number of NOE restraints perform differently in driving the structure determination to the correct fold. For every given set of restraints, 200 structure models were computed with and without the sPRE data. To quantify the impact of the sPRE data, the 20 best models of each run (10%, scored according to the total energy) were selected and the RMSD to the published NMR structures was computed. Computation of RMSD was performed on all carbon, nitrogen and phosphorus atoms in the case of the UUCG tetraloop. For the GTP-bound aptamer, the RMSD was computed using all carbon, nitrogen and phosphorus atoms of the GTP ligand and all non-terminal nucleotides except the intrinsically flexible nucleotides A13 and U21^40^.

A second benchmark was performed to address the performance of the sPRE potential in the absence of any other experimental restraint as well as in combination with other experimental NMR restraints, such hydrogen bonds, NOEs, RDCs and torsion angles. Experimental restraints for the UUCG tetraloop were obtained from PDB entry 2KOC^37, 38^. Two hundred structure models of the UUCG tetraloop were computed with and without the sPRE data for every restraint set (compare Supplementary Tables 5) and the 20 best scored models according to the total energy were selected. The average RMSD to the published NMR structure was computed for the top 20 models and used to quantify the impact of the sPRE data (computation of RMSD was performed on all carbon, nitrogen and phosphorus atoms).

### Data Availability

The datasets generated during and/or analysed during the current study are available from the corresponding author on reasonable request. All scripts used for data analysis and back-calculation of sPRE data are openly available (http://mbbc.medunigraz.at/forschung/forschungseinheiten-und-gruppen/forschungsgruppe-tobias-madl/software/).

## Electronic supplementary material

Supplementary Information
