# Supplementary material for: RNA structure refinement using NMR solvent accessibility data
Source: Sci Rep. 2017 Jul 14;7:5393. doi: 10.1038/s41598-017-05821-z (PMC5511288; doi:10.1038/s41598-017-05821-z)
Supplement: Supplementary file 1 — Supplementary Information [file 41598_2017_5821_MOESM1_ESM.pdf]

Supplementary Information

RNA structure refinement using NMR solvent accessibility data

Christoph Hartlmüller[1,2] *, Johannes C. Günther[1,2] *, Antje C. Wolter[3], Jens Wöhnert[3], Michael Sattler[1,2] & Tobias Madl[1,2,4]

[1]Center for Integrated Protein Science Munich, Department Chemie, Technical University of Munich, Lichtenbergstr. 4, 85748 Garching, Germany. [2]Institute of Structural Biology, Helmholtz Zentrum München, Ingolstadter Landstr. 1, 85764 Neuherberg, Germany. [3]Institut für Molekulare Biowissenschaften and Zentrum für Biomolekulare Magnetische Resonanz (BMRZ), Goethe-Universität Frankfurt, Max-von-Laue Str. 9, 60438 Frankfurt/M, Germany. [4]Institute of Molecular Biology and Biochemistry, Center of Molecular Medicine, Medical University of Graz, Har-rachgasse 21, 8010 Graz, Austria. * These authors contributed equally to this work. Correspondence and requests for materials should be addressed to T.M. (email: tobias.madl@medunigraz.at).

Supplementary Figures

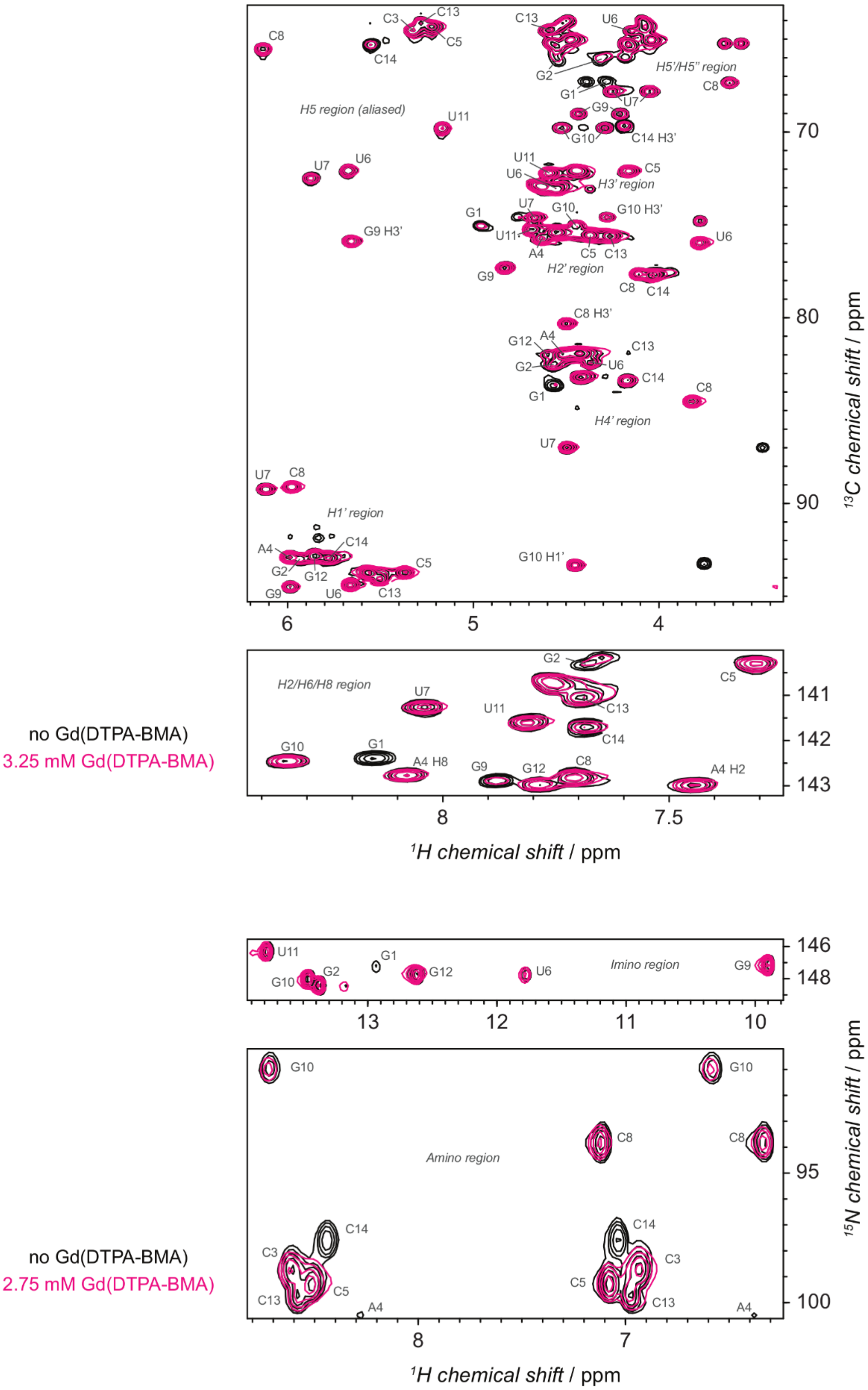

Supplementary Figure 1 | NMR spectra of the UUCG tetraloop in the absence (black) and presence of paramagnetic compound (magenta). The shown regions of the spectra are (from top to bottom) the sugar (including H5), the bases (H6 and H8), the imino and the amino region. Note that some resonances appear at the aliased frequency. Assignments were obtained from BMRB entry 5705[1].

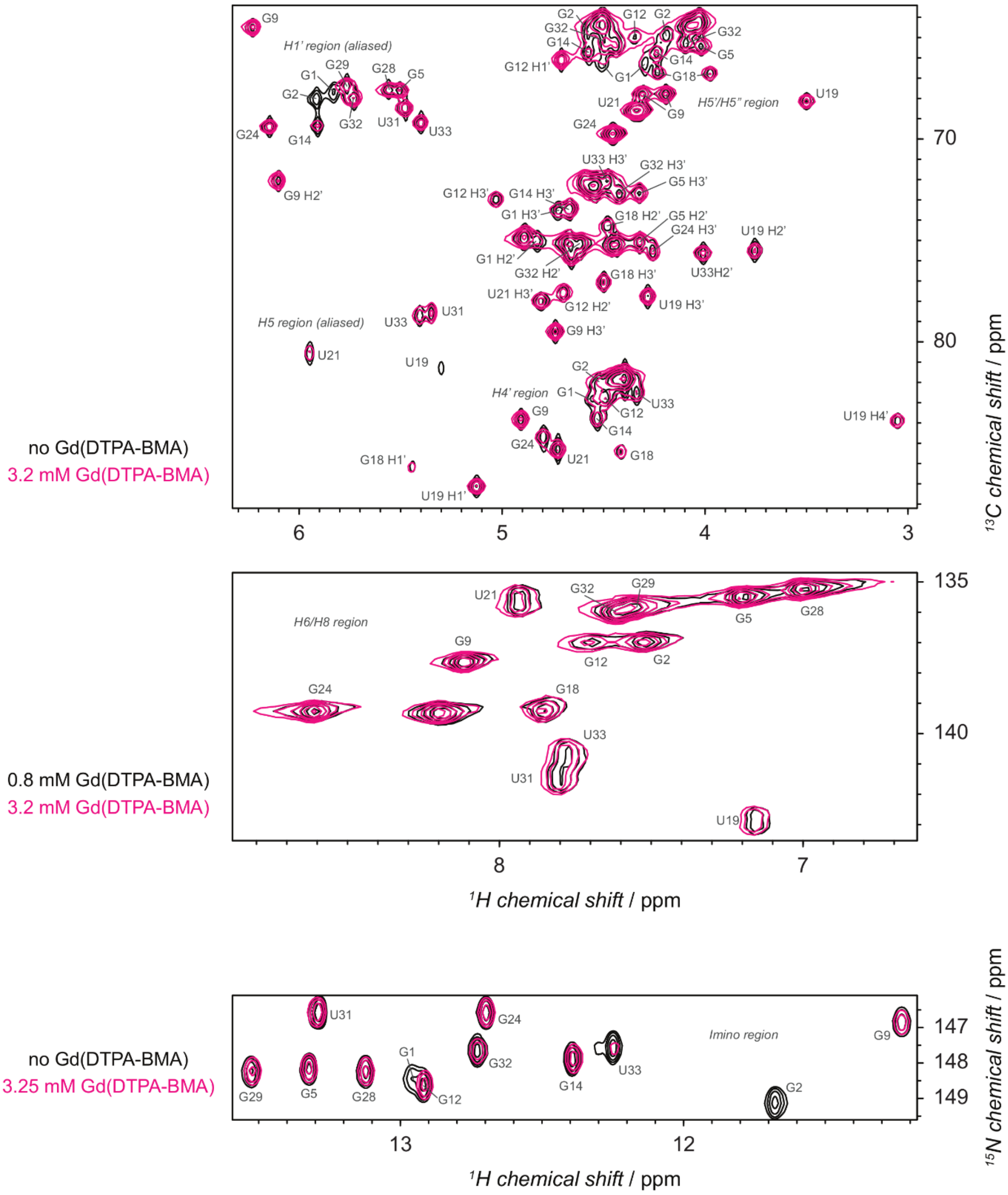

Supplementary Figure 2 | NMR spectra of a (^{13}C, ^{15}N)-GU labeled sampled of the GTP aptamer in the absence (black) and presence of paramagnetic compound (magenta). The shown regions of the spectra are (from top to bottom) the sugar (including H5), the bases (H6 and H8) and the imino region. Note that some resonances appear at the aliased frequency. Assignments were obtained from BMRB entry 25661[2].

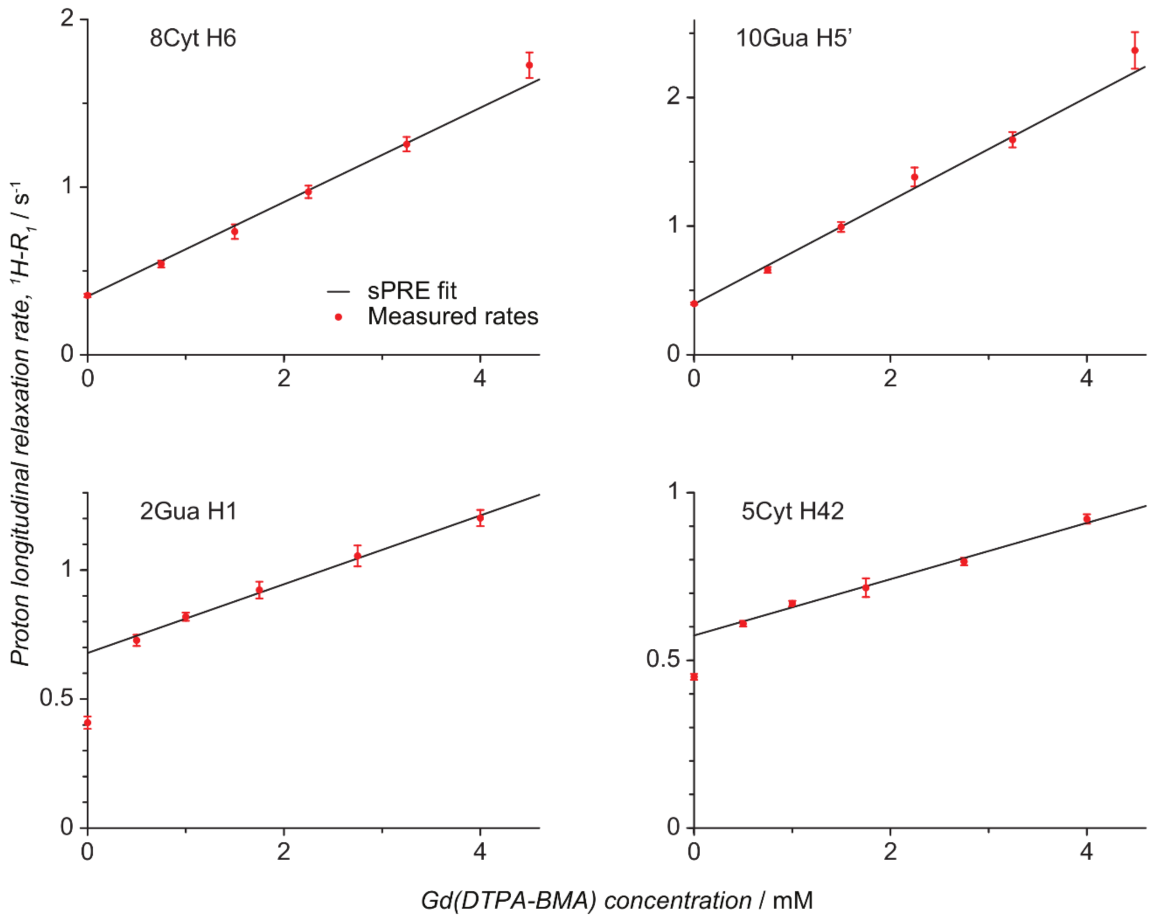

Supplementary Figure 3 | Increase of proton R_1 as function of the concentration of Gd(DTPA-BMA). Rates are plotted as red dots for carbon-bound (8Cyt H6 and 10Gua H5’) as well as for nitrogen-bound (2Gua H1 and 5Cyt H42) protons of the UUCG tetraloop. The parameters obtained from fitting the linear sPRE model are shown as a black line. For the nitrogen-bound protons, R_1 data points in the absence of paramagnetic compound were not used in the weighted linear regression to fit the sPRE model (compare Supplementary Table 1).

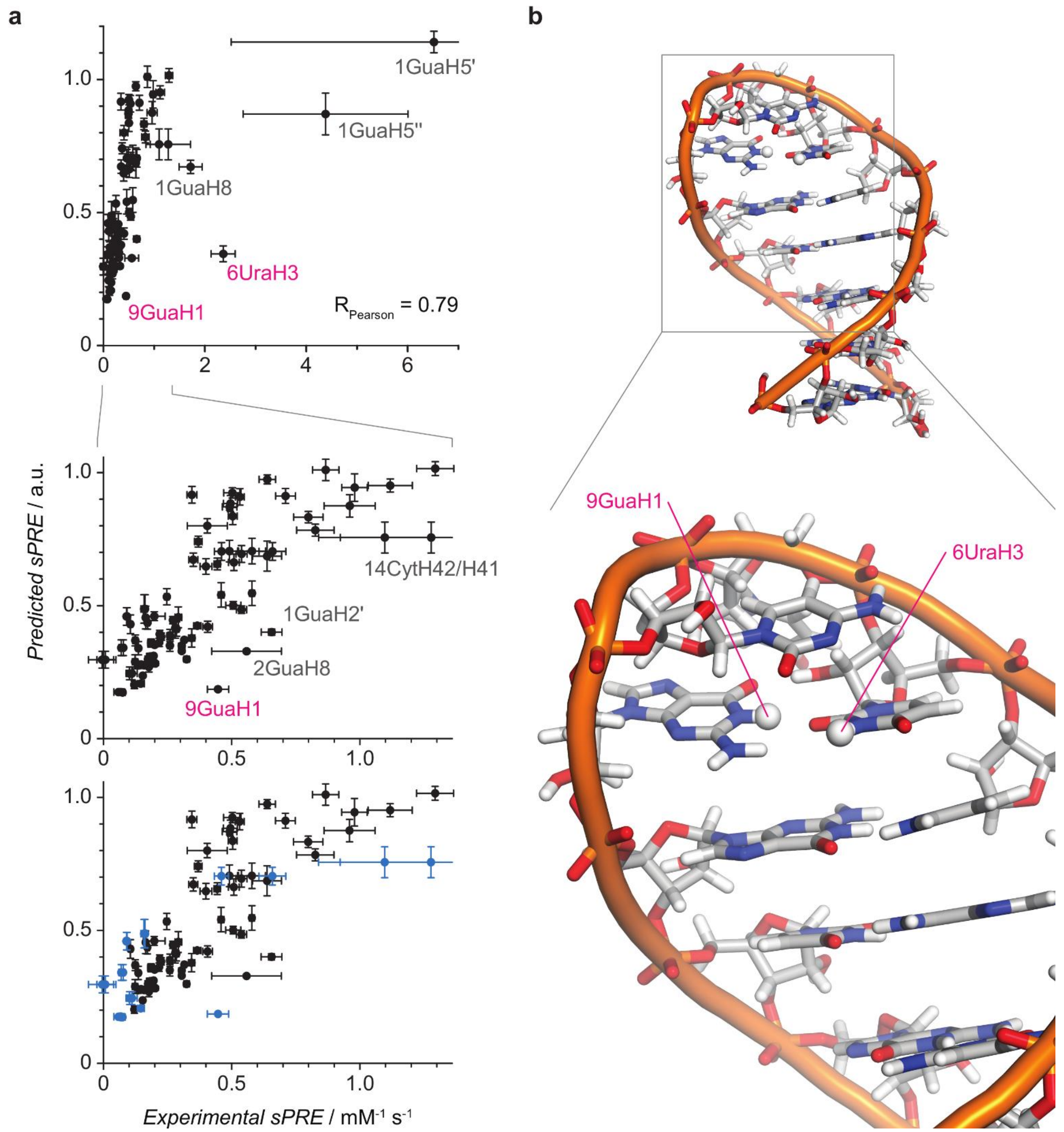

Supplementary Figure 4 | sPRE data of the UUCG tetraloop correlate well with the corresponding structure. (**a**) Correlation of experimental and predicted sPRE data for the UUCG tetraloop is shown. The middle panel is an expansion of the top panel. Outliers located at terminal nucleotides are labeled in grey and those located in the loop region are labeled in magenta. In the bottom plot, the carbon-bound protons are shown in black and the nitrogen-bound protons are drawn in blue. The Pearson correlation coefficient was calculated using all data points for which the errors of the experimental and predicted sPRE value is below 10 %. (**b**) The complete structural model of the UUCG tetraloop (PDB code 2KOC) is shown on the top and a close-up of the loop region is shown on the bottom. The positions of the outliers are indicated and both protons are shown as larger spheres.

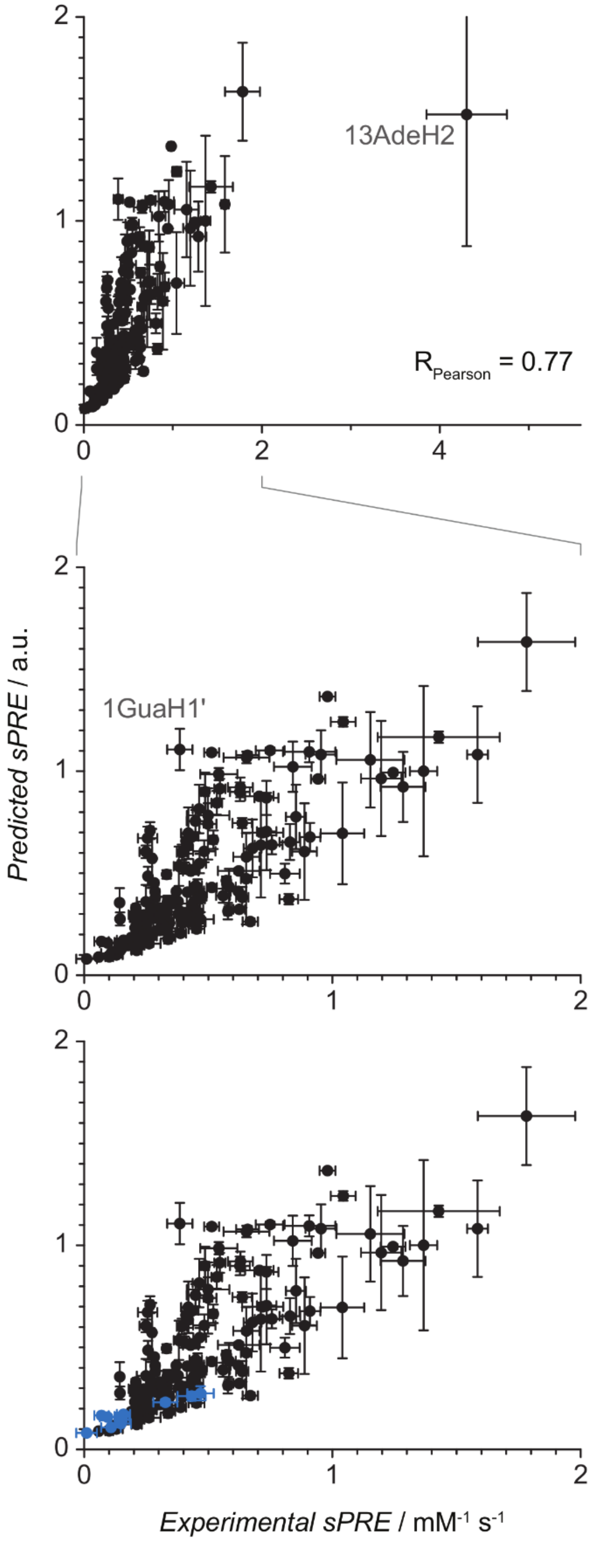

Supplementary Figure 5 | sPRE data of the GTP-bound GTP class II aptamer correlate well with the corresponding structure. The correlation of experimental and predicted sPRE data for the ligand-bound GTP aptamer is shown and outliers are labeled in grey. In the bottom plot, the carbon-bound protons are shown in black and the nitrogen-bound protons are drawn in blue. The Pearson correlation coefficient was calculated using all data points for which the errors of the experimental and predicted sPRE value is below 10 %.

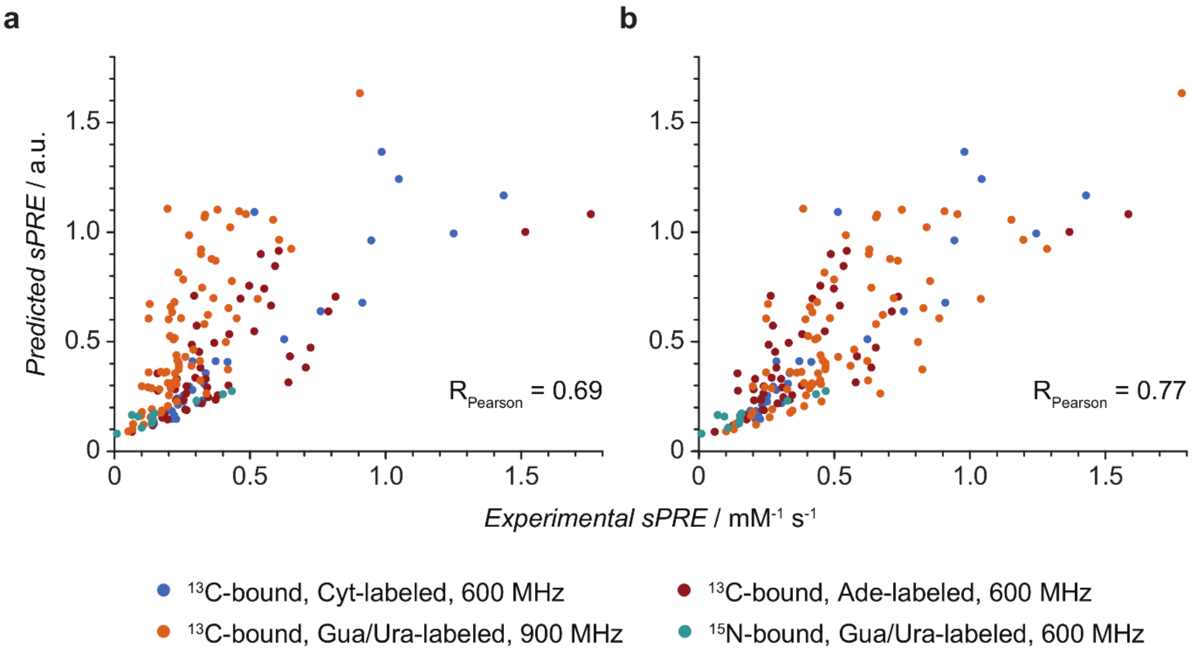

Supplementary Figure 6 | Effect of normalization using the sPRE of the water solvent. Scatter plots show the measured and predicted sPRE data of the GTP-bound GTP aptamer before (**a**) and after (**b**) normalization of the experimental sPRE data. The data was obtained in several experiments (using 3 different labeling schemes, H_2O and D_2O-based buffer and two different field strengths) as indicated by the different colors. For each of the experiments, the sPRE of water solvent was measured and used to normalize the sPRE. The normalized data sets were then rescaled by the average sPRE of water solvent in all experiments. The Pearson correlation coefficients were calculated using all data points for which the errors of the experimental and predicted sPRE value is below 10 %. sPRE data for carbon- and nitrogen-bound protons were acquired in D_2O and H_2O buffer, respectively.

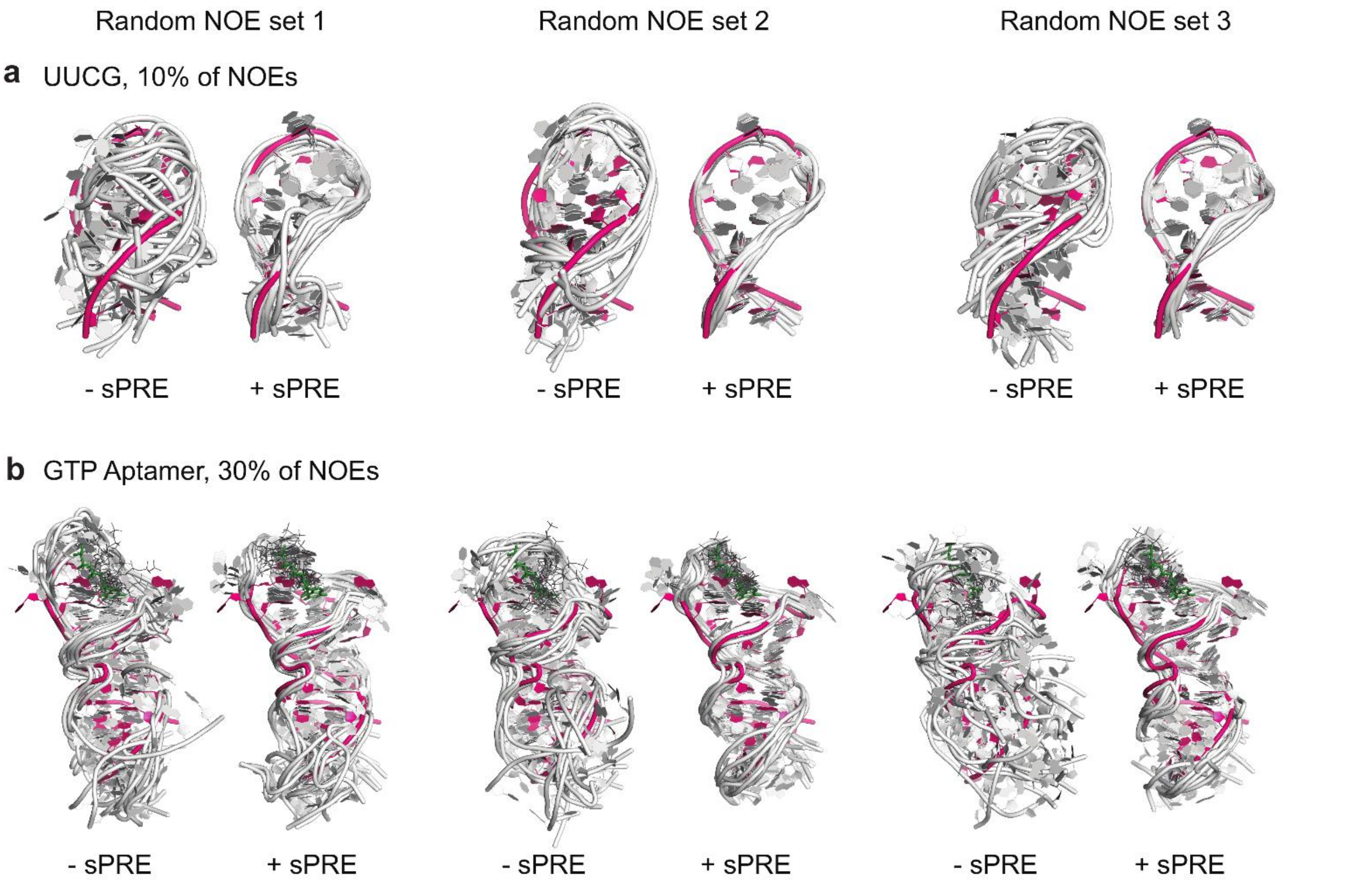

Supplementary Figure 7 | sPRE data improve structure determination of RNAs with sparse data sets. Structural models of the UUCG tetraloop (a) and the GTP-bound GTP aptamer (b) were obtained without (-sPRE) and with (+sPRE) sPRE data using XplorNIH (see also supplementary tables 3 and 4). The 10 best scored models (light gray) in terms of total energy were selected from a total of 200 models and aligned to the corresponding NMR structure (magenta). All restraints used in the computations are indicated. In (b) heavy atoms of the GTP ligand are shown as sticks (Reference in green, computed models in dark gray).

Supplementary Tables

Supplementary Table 1 | Experimental details for the acquisition of sPRE data for the UUCG tetraloop. All experiments were acquired on a 900 MHz spectrometer equipped with a cryo probe head.

^{15}N amino region

Conc. of Gd(DTPA-BMA) [mM]	Number of delays	Longest delay [s]	Number of scans	Number of increments	Approx. NMR time
0 [a]	12	6	8	20	1:38 h
0.5	12	6	16	20	3:16 h
1	14	6	16	20	3:40 h
1.75	12	4	16	20	2:11 h
2.75	12	3.5	20	20	2:24 h
4	12	3	28	20	2:54 h

[a] Not used for fitting the linear sPRE model

^{15}N imino region

Conc. of Gd(DTPA-BMA) [mM]	Number of delays	Longest delay [s]	Number of scans	Number of increments	Approx. NMR time
0 [a]	12	6	16	6	0:59 h
0.5	12	6	20	6	1:13 h
1	14	6	20	6	1:22 h
1.75	12	4	20	6	0:49 h
2.75	12	3.5	24	6	0:52 h
4	12	3	36	6	1:07 h

[a] Not used for fitting the linear sPRE model

^{13}C sugar region

Conc. of Gd(DTPA-BMA) [mM]	Number of delays	Longest delay [s]	Number of scans	Number of increments	Approx. NMR time
0	9	11	8	81	9:17 h
0.75	9	8	8	80	6:41 h
1.5	9	5.5	8	80	4:37 h
2.25	11	5	8	80	3:55 h
3.25	11	4	8	80	3:08 h
4.5	11	4.5	8	80	3:31 h

^{13}C base region

Conc. of Gd(DTPA-BMA) [mM]	Number of delays	Longest delay [s]	Number of scans	Number of increments	Approx. NMR time
0	9	10	16	19	3:57 h
0.75	9	8	16	19	3:10 h
1.5	9	5.5	16	19	2:11 h
2.25	11	5	16	19	1:51 h
3.25	11	5	16	19	1:51 h
4.5	11	4.5	16	19	1:40 h

Supplementary Table 2 | Experimental details for the acquisition of sPRE data for the GTP aptamer.

^{15}N imino region, GU-labeled sample, 600 MHz spectrometer with cryo probe head

Conc. of Gd(DTPA-BMA) [mM]	Number of delays	Longest delay [s]	Number of scans	Number of increments	Approx. NMR time
0 [a]	22	7	48	6	5:43 h
0.645 [a]	22	7	56	6	6:40 h
1.29	22	6	56	6	5:43 h
1.94	22	4	72	6	4:56 h
2.58	22	3	72	6	3:43 h
3.23	22	3	100	6	5:09 h

[a] Not used for fitting the linear sPRE model

^{13}C sugar region, GU-labeled sample, 900 MHz spectrometer with cryo probe head

Conc. of Gd(DTPA-BMA) [mM]	Number of delays	Longest delay [s]	Number of scans	Number of increments	Approx. NMR time
0	11	10	40	50	24:20 h
0.8	11	8	40	50	19:28 h
1.6	11	6	42	50	15:23 h
2.4	11	5.5	44	50	14:47 h
3.2	11	5	44	50	13:28 h
4	11	4.5	42	50	11:35 h

^{13}C base region, GU-labeled sample, 900 MHz spectrometer with cryo probe head

Conc. of Gd(DTPA-BMA) [mM]	Number of delays	Longest delay [s]	Number of scans	Number of increments	Approx. NMR time
0	11	10	40	20	9:43 h
0.8	11	8	38	20	7:23 h
1.6	11	6	38	20	5:33 h
2.4	11	5	38	20	4:38 h
3.2	11	5	38	20	4:38 h
4	11	4.5	34	20	3:44 h

^{13}C sugar region, A-labeled sample, 600 MHz spectrometer with cryo probe head

Conc. of Gd(DTPA-BMA) [mM]	Number of delays	Longest delay [s]	Number of scans	Number of increments	Approx. NMR time
0	11	8.5	30	40	16:23 h
0.8	11	6	36	40	13:55 h
1.6	11	4.75	50	40	15:21 h
2.4	11	3.5	66	40	15:00 h
3.2	17	3	92	40	25:07 h
4	11	2.5	80	40	13:06 h

^{13}C base region, A-labeled sample, 600 MHz spectrometer with cryo probe head

Conc. of Gd(DTPA-BMA) [mM]	Number of delays	Longest delay [s]	Number of scans	Number of increments	Approx. NMR time
0	11	8.5	38	15	7:47 h
0.8	11	6	38	15	5:30 h
1.6	11	6	56	15	6:09 h
2.4	17	6	56	15	8:23 h
3.2	17	6	60	15	8:59 h
4	17	4	50	15	5:01 h

^{13}C sugar region, C-labeled sample, 600 MHz spectrometer with cryo probe head

Conc. of Gd(DTPA-BMA) [mM]	Number of delays	Longest delay [s]	Number of scans	Number of increments	Approx. NMR time
0	11	10	56	25	22:27 h
0.8	11	7.5	50	32	19:18 h
1.6	11	4.5	58	32	13:30 h
2.4	11	3.5	66	32	12:00 h
3.2	11	2.5	74	32	13:31 h

4	11	2.5	74	32	7:23 h
4.8	11	2.5	64	32	6:23 h

^{13}C base region, C-labeled sample, 600 MHz spectrometer with cryo probe head

Conc. of Gd(DTPA-BMA) [mM]	Number of delays	Longest delay [s]	Number of scans	Number of increments	Approx. NMR time
0	11	10	56	8	7:11 h
0.8	11	7.5	64	8	6:10 h
1.6	11	4.5	70	8	4:04 h
2.4	11	3.5	80	8	3:38 h
3.2	11	2	80	8	2:06 h
4	11	2	80	8	1:36 h
4.8	11	2	72	8	1:27

Supplementary Table 3 | sPRE data improves structure determination of the UUCG tetraloop. Structural models of the UUCG tetraloop were computed using hydrogen bonds-derived restraints in combination with different sets of experimental NOEs obtained from PDB entry 2KOC. The size of the NOE restraints set was varied and for every size, 3 randomly created sets were generated. For every restraint set, 200 models were computed in the absence and presence of the sPRE potential and the best 20 models based on the total energy were selected. RMSD values are computed by comparing all carbon, nitrogen and phosphorus atoms to the published NMR structure and the average RMSDs of the 20 best scored models are shown in the table.

Number of NOE restraints (percentage of all unambig. NOEs)	sPRE data used	Average RMSD of best scored models [Å]		
		Random set 1	Random set 2	Random set 3
25 (10%)	no	7.32	4.75	5.80
25 (10%)	yes	2.10	1.35	1.77
50 (20%)	no	3.49	6.57	2.03
50 (20%)	yes	1.39	1.49	1.41
75 (30%)	no	1.90	2.64	4.52
75 (30%)	yes	1.58	1.39	1.60
100 (40%)	no	1.94	1.88	1.54
100 (40%)	yes	1.30	1.30	1.46
125 (50%)	no	1.56	1.31	1.28
125 (50%)	yes	1.23	1.39	1.13
188 (75%)	no	1.44	1.27	1.05
188 (75%)	yes	1.05	1.28	1.04
251 (100%)	no	1.13		
251 (100%)	yes	1.05		

Supplementary Table 4 | sPRE data improves structure determination of the GTP class II aptamer in complex with GTP. Structural models of the ligand-bound aptamer were computed using experimental hydrogen bonds-derived restraints in combination with different sets of experimental NOEs. The size of the NOE restraints set was varied and for every size, at least 3 randomly created sets were generated. For every restraint set, 200 models were computed in the absence and presence of the sPRE potential and the best 20 models based on the total energy were selected. RMSD values are computed by comparing all carbon, nitrogen and phosphorus atoms of the GTP ligand and all non-terminal nucleotides (except the flexible A13 and U21) to the published NMR structure. The average RMSDs of the 20 best scored models are shown in the table.

Number of NOE restraints (percentage of all NOEs)	sPRE data used	Average RMSD of best scored models [Å]				
		Random set 1	Random set 2	Random set 3	Random set 4	Random set 5
43 (5%)	no	11.31	11.44	10.77		
43 (5%)	yes	8.61	6.98	7.79		
86 (10%)	no	10.24	10.13	10.71	11.30	9.92
86 (10%)	yes	6.44	5.45	5.21	5.69	5.96
172 (20%)	no	7.90	9.06	9.64		
172 (20%)	yes	5.27	5.35	6.11		
258 (30%)	no	6.54	7.80	9.56		
258 (30%)	yes	5.96	3.51	4.01		
345 (40%)	no	4.49	4.56	5.13		
345 (40%)	yes	3.44	2.96	4.71		
431 (50%)	no	3.55	4.55	3.77		
431 (50%)	yes	2.44	3.46	2.61		
647 (75%)	no	3.39	3.40	2.84		
647 (75%)	yes	2.33	2.78	2.81		
863 (100%)	no	2.30				
863 (100%)	yes	2.18				

Supplementary Table 6 | sPRE data is an orthogonal restraint that can be combined with classical NMR restraints. Structural models of the UUCG tetraloop were computed in the absence and presence of the sPRE potential and in combination with different experimental NMR restraints obtained from PDB entry 2KOC. For every restraint set, 200 models were computed and the best 20 models based on the total energy were selected. RMSD values are computed by comparing all carbon, nitrogen and phosphorus atoms to the published NMR structure and the average RMSDs of the 20 best scored models are shown in the table.

Restraints used	sPRE data used	Average RMSD of best scored models [Å]
Force field only	no sPRE	12.49
	with sPRE data	8.69
Hydrogen bonds	no sPRE	7.77
	with sPRE data	2.83
Hydrogen bonds + RDCs	no sPRE	6.90
	with sPRE data	2.62
Hydrogen bonds + Torsion angles	no sPRE	1.75
	with sPRE data	1.28
Hydrogen bonds + all NOEs (ambig. and unambig.)	no sPRE	1.06
	with sPRE data	1.05
Hydrogen bonds + Torsion angles + RDCs + all NOEs (ambig. and unambig.)	no sPRE	0.76
	with sPRE data	0.79

Supplementary Table 6 | Sparse sPRE data sets improve structure determination of the UUCG tetraloop. Structural models of the UUCG tetraloop were computed using hydrogen bonds-derived restraints in combination with different sets of synthetic sPREs obtained from PDB entry 2KOC using the back-calculation as described in methods. The size of the sPRE restraints set was varied and for every size, 5 randomly created sets were generated. For every restraint set, 200 models were computed in the presence of the sPRE potential and the best 20 models based on the total energy were selected. RMSD values are computed by comparing all carbon, nitrogen and phosphorus atoms to the published NMR structure and the average RMSDs of the 20 best scored models are shown in the table. Note that the result obtained for 97 experimental sPREs (Supplementary Table 6, RMSD = 2.83 Å) and 101 synthetic sPREs (this table, 2.75 Å) is comparable indicating that our approach performs equally well with experimental and synthetic sPRE data.

Percentage of sPRE restraints [%]	Number of sPRE restraints	Average RMSD of best scored models [Å]				
		Random set 1	Random set 2	Random set 3	Random set 4	Random set 5
6.25	6	7.50	7.87	7.03	7.03	7.55
12.5	13	6.46	7.64	6.64	7.46	6.19
25	25	7.93	4.93	4.30	7.54	6.68
50	50	3.58	3.41	3.92	3.99	3.09
75	76	3.38	2.87	3.34	3.07	3.14
100	101	2.75				

XplorNIH Protocols

UUCG tetraloop

```
# protocol is based on gb1_rdc example

xplor.requireVersion("2.24")

xplor.parseArguments()

import os
import sys, traceback
import protocol
output_folder="output"
outFilename = os.path.join(output_folder, "SCRIPT_STRUCTURE.sa")

numberOfStructures=200
protocol.initRandomSeed()
command = xplor.command

# load RNA topology
protocol.topology['nucleic'] = "nucleic-3.1_GTP_AP7.top"
protocol.parameters['nucleic'] = "nucleic-3.1_GTP_AP7.par"

from psfGen import seqToPSF
xplor.command('''
 topology
    @TOPPAR:nucleic-3.1_GTP_AP7.top
 end
 parameter
    @TOPPAR:nucleic-3.1_GTP_AP7.par
 end

 segment                                   {*Generate protein              *}
   name="    "                             {*This name has to match the    *}
                                           {*four characters in columns 73 *}
                                           {*through 76 in the coordinate  *}
                                           {*file, in XPLOR this name is   *}
                                           {*name is referred to as SEGId. *}
   chain
     @TOPPAR:toph11.nuc                    {*Read peptide bond file        *}
     sequence ''' +
# sequence file for RNA
open("UUCG.seq").read() +
     ''' end           {*interpret sequence file to  *}
   end                                     {*obtain the sequence           *}
 end
''')


protocol.genExtendedStructure()

from potList import PotList
potList = PotList()

from simulationTools import MultRamp, StaticRamp, InitialParams

rampedParams=[]
highTempParams=[]

from posDiffPotTools import create_PosDiffPot
refRMSD = create_PosDiffPot("refRMSD",
                            "(name P* or name N* or name C*) and not (resi 1 and name P*)",
                            pdbFile='2KOC__model1.pdb' )


noe=PotList('noe')
potList.append(noe)
from noePotTools import create_NOEPot
for (name,scale,file) in [('noes',1,"noe_2koc.tbl"),
                          ('hbonds',1,"hbond_2koc.tbl")
                          ]:
    pot = create_NOEPot(name,file)
    pot.setScale(scale)
    noe.append(pot)
rampedParams.append( MultRamp(2,30, "noe.setScale( VALUE )") )


# create Potential for sPRE
import nbTargetPotTools
spre_pot = nbTargetPotTools.create_NBTargetPot("spre", restraints=open("UUCG_sPRE.tbl").read() , selection='all', restraintFormat="xplor")
spre_pot.setCutoffDist(20)
spre_pot.setAveType("center")      # sum or center
spre_pot.setPotType("correlation")    # rmsd or correlation
spre_pot.setAveExp(6)           # 6 is the default anyway
spre_pot.setInvPow(2)           # 2 is the default

# set intercept and slope obtained from calibrate function
spre_pot.setIntercept(-0.257261)
spre_pot.setSlope(2.25292)
potList.append(spre_pot)
rampedParams.append( MultRamp(1000,1000, "spre_pot.setScale( VALUE )") )
```

```
from varTensorTools import create_VarTensor
media={}
for (medium,Da,Rh) in [ ('medium1',   7.5, 0.5) ]:
    oTensor = create_VarTensor(medium)
    oTensor.setDa(Da)
    oTensor.setRh(Rh)
    oTensor.setFreedom("varyDa, varyRh")
    media[medium] = oTensor
    pass

from rdcPotTools import create_RDCPot, scale_toCH
rdcs = PotList('rdc')
for (medium,expt,file,weight) in     [
     ('medium1','CH','rdc-CH_2koc.tbl' ,1)
     ]:
    rdc = create_RDCPot("%s_%s"%(medium,expt),file,media[medium])

    rdc.setScale(weight)
    if expt != 'CH':
        scale_toCH(rdc)
    rdcs.append(rdc)
    pass
potList.append(rdcs)
rampedParams.append( MultRamp(0.01,1.0, "rdcs.setScale( VALUE )") )


from xplorPot import XplorPot
xplor.command("@plane_2koc.tbl")
potList.append(XplorPot("plan",xplor.simulation))


dihedralRestraintFilename="torsion_2koc.tbl"
protocol.initDihedrals(dihedralRestraintFilename)
potList.append( XplorPot('CDIH') )
highTempParams.append( StaticRamp("potList['CDIH'].setScale(10)") )
rampedParams.append( StaticRamp("potList['CDIH'].setScale(200)") )
potList['CDIH'].setThreshold( 5 )


# use new torsion potential RNA-ff1
#   Bermejo, G. A.; Clore, G. M.; Schwieters, C. D. Structure 2016, 24, 806
import torsionDBPotTools
torsiondb = torsionDBPotTools.create_TorsionDBPot(name='torsiondb',database='rna09_v0.dat')
potList.append(torsiondb)
rampedParams.append(MultRamp(0.1, 1, "torsiondb.setScale(VALUE)"))


potList.append( XplorPot('VDW') )
rampedParams.append( StaticRamp("protocol.initNBond()") )
rampedParams.append( MultRamp(1.0,0.9,
                              "command('param nbonds repel VALUE end end')") )
rampedParams.append( MultRamp(.004,4,
                              "command('param nbonds rcon VALUE end end')") )
highTempParams.append( StaticRamp("""protocol.initNBond(cutnb=100,
                                                         rcon=0.004,
                                                         tolerance=45,
                                                         repel=1.2,
                                                         selStr="name C1'")""") )
potList.append( XplorPot("BOND") )
potList.append( XplorPot("ANGL") )
potList['ANGL'].setThreshold( 5 )
rampedParams.append( MultRamp(0.4,1,"potList['ANGL'].setScale(VALUE)") )
potList.append( XplorPot("IMPR") )
potList['IMPR'].setThreshold( 5 )
rampedParams.append( MultRamp(0.1,1,"potList['IMPR'].setScale(VALUE)") )

protocol.massSetup()

from ivm import IVM
dyn = IVM()

protocol.torsionTopology(dyn)

minc = IVM()
protocol.initMinimize(minc)

protocol.cartesianTopology(minc)

from simulationTools import AnnealIVM
init_t  = 3500.
cool = AnnealIVM(initTemp =init_t,
                 finalTemp=25,
                 tempStep =12.5,
                 ivm=dyn,
                 rampedParams = rampedParams)

scale_anneal_time = 10

def calcOneStructure(loopInfo):
    """ this function calculates a single structure, performs analysis on the
    structure, and then writes out a pdb file, with remarks.
    """
    from monteCarlo import randomizeTorsions
```

```
    randomizeTorsions(dyn)
    try:
        protocol.fixupCovalentGeom(maxIters=100, useVDW=1)
    except protocol.CovalentViolation:
        pass


    protocol.writePDB(loopInfo.filename()+".init")

    InitialParams( rampedParams )

    InitialParams( highTempParams )

    protocol.initDynamics(dyn,
                          potList=potList,
                          bathTemp=init_t,
                          initVelocities=1,
                          finalTime=100*scale_anneal_time,
                          numSteps=1000*scale_anneal_time,
                          printInterval=100)

    dyn.setETolerance( init_t/100 )
    dyn.run()

    InitialParams( rampedParams )

    protocol.initDynamics(dyn,
                          potList=potList,
                          numSteps=200*scale_anneal_time,
                          finalTime=.4*scale_anneal_time,
                          printInterval=100)

    cool.run()

    protocol.initMinimize(dyn, printInterval=50)
    dyn.run()

    protocol.initMinimize(minc,
                          potList=potList,
                          dEPred=10)
    minc.run()


from simulationTools import StructureLoop, FinalParams
StructureLoop(numStructures=numberOfStructures,
              doWriteStructures=True,
              pdbTemplate=outFilename,
              structLoopAction=calcOneStructure,
              genViolationStats=True,
              averageTopFraction=0.5,
              averageSortPots=[potList['BOND'],potList['ANGL'],potList['IMPR'],noe,rdcs],
              averageContext=FinalParams(rampedParams),
              averageCrossTerms=refRMSD,
              averageFilename="SCRIPT_ave.pdb",
              averagePotList=potList).run()
```

GTP-bound aptamer

```
# protocol is based on gb1_rdc example

xplor.requireVersion("2.24")

xplor.parseArguments()

import os
import sys, traceback
import protocol
output_folder="output"
outFilename = os.path.join(output_folder, "SCRIPT_STRUCTURE.sa")

numberOfStructures=200
protocol.initRandomSeed()   #set random seed - by time
command = xplor.command

# load RNA topology
protocol.topology['nucleic'] = "nucleic-3.1_GTP_AP7.top"
protocol.parameters['nucleic'] = "nucleic-3.1_GTP_AP7.par"

from psfGen import seqToPSF
xplor.command('''
 topology
    @TOPPAR:nucleic-3.1_GTP_AP7.top
 end
 parameter
    @TOPPAR:nucleic-3.1_GTP_AP7.par
 end

 segment                                  {*Generate protein             *}
   name="    "                            {*This name has to match the    *}
                                          {*four characters in columns 73 *}
                                          {*through 76 in the coordinate  *}
                                          {*file, in XPLOR this name is   *}
                                          {*name is referred to as SEGId. *}
   chain
     @TOPPAR:toph11.nuc                   {*Read peptide bond file        *}
     sequence ''' +
# sequence file for RNA
open("GTP_aptamer.seq").read() +
     ''' end           {*interpret sequence file to  *}
   end                                    {*obtain the sequence           *}
 end
segment
   name="    "                            {*This name has to match the    *}
                                          {*four characters in columns 73 *}
                                          {*through 76 in the coordinate  *}
                                          {*file, in XPLOR this name is   *}
                                          {*name is referred to as SEGId. *}

   number=99                              {*Residue number                *}


   chain
      sequence GTP end
   end
end
''')



protocol.genExtendedStructure()

from potList import PotList
potList = PotList()

from simulationTools import MultRamp, StaticRamp, InitialParams

rampedParams=[]
highTempParams=[]

from posDiffPotTools import create_PosDiffPot
refRMSD = create_PosDiffPot("refRMSD",
                            "(name P* or name N* or name C*) and not (resi 1 and name P*)",
                            pdbFile='Reference_aptamer__model1.pdb' )


noe=PotList('noe')
potList.append(noe)
from noePotTools import create_NOEPot
for (name,scale,file) in [('noes',1,"noe_list_xplor.tbl"),
                          ('hbonds',1,"hbond_list_xplor.tbl"),
                          ('lowerlimits',1,"noe_lol_list_xplor.tbl")
                          ]:
    pot = create_NOEPot(name,file)
    pot.setScale(scale)
    noe.append(pot)
rampedParams.append( MultRamp(2,30, "noe.setScale( VALUE )") )


# create Potential for sPRE
import nbTargetPotTools
```

```
spre_pot = nbTargetPotTools.create_NBTargetPot("spre", restraints=open("GTPaptamer_sPRE.tbl").read() , selection='all',
restraintFormat="xplor")
spre_pot.setCutoffDist(20)
spre_pot.setAveType("center")      # sum or center
spre_pot.setPotType("correlation")     # rmsd or correlation
spre_pot.setAveExp(6)           # 6 is the default anyway
spre_pot.setInvPow(2)           # 2 is the default

# set intercept and slope obtained from calibrate function
spre_pot.setIntercept(-0.157217)
spre_pot.setSlope(3.25629)
potList.append(spre_pot)
rampedParams.append( MultRamp(3000,3000, "spre_pot.setScale( VALUE )") )



from xplorPot import XplorPot
xplor.command("@plane.inp")
potList.append(XplorPot("plan",xplor.simulation))


# use new torsion potential RNA-ff1
#   Bermejo, G. A.; Clore, G. M.; Schwieters, C. D. Structure 2016, 24, 806
import torsionDBPotTools
torsiondb = torsionDBPotTools.create_TorsionDBPot(name='torsiondb',database='rna09_v0.dat')
potList.append(torsiondb)
rampedParams.append(MultRamp(0.3, 0.3, "torsiondb.setScale(VALUE)"))


potList.append( XplorPot('VDW') )
rampedParams.append( StaticRamp("protocol.initNBond()") )
rampedParams.append( MultRamp(1.0,0.9,
                              "command('param nbonds repel VALUE end end')") )
rampedParams.append( MultRamp(.004,4,
                              "command('param nbonds rcon VALUE end end')") )
# nonbonded interaction only between CA atoms
highTempParams.append( StaticRamp("""protocol.initNBond(cutnb=100,
                                                        rcon=0.004,
                                                        tolerance=45,
                                                        repel=1.2,
                                                        selStr="name C1'")""") )


potList.append( XplorPot("BOND") )
potList.append( XplorPot("ANGL") )
potList['ANGL'].setThreshold( 5 )
rampedParams.append( MultRamp(0.4,1,"potList['ANGL'].setScale(VALUE)") )
potList.append( XplorPot("IMPR") )
potList['IMPR'].setThreshold( 5 )
rampedParams.append( MultRamp(0.1,1,"potList['IMPR'].setScale(VALUE)") )

protocol.massSetup()

from ivm import IVM
dyn = IVM()

protocol.torsionTopology(dyn)

minc = IVM()
protocol.initMinimize(minc)

protocol.cartesianTopology(minc)

from simulationTools import AnnealIVM
init_t  = 3500.
cool = AnnealIVM(initTemp =init_t,
                 finalTemp=25,
                 tempStep =12.5,
                 ivm=dyn,
                 rampedParams = rampedParams)

scale_anneal_time = 10

def calcOneStructure(loopInfo):
    """ this function calculates a single structure, performs analysis on the
    structure, and then writes out a pdb file, with remarks.
    """
    from monteCarlo import randomizeTorsions
    randomizeTorsions(dyn)
    try:
        protocol.fixupCovalentGeom(maxIters=100, useVDW=1)
    except protocol.CovalentViolation:
        pass


    protocol.writePDB(loopInfo.filename()+".init")

    InitialParams( rampedParams )

    InitialParams( highTempParams )

    protocol.initDynamics(dyn,
                            potList=potList,
                            bathTemp=init_t,
                            initVelocities=1,
```

```
                        finalTime=100*scale_anneal_time,
                        numSteps=1000*scale_anneal_time,
                        printInterval=100)

    dyn.setETolerance( init_t/100 )
    dyn.run()

    InitialParams( rampedParams )

    protocol.initDynamics(dyn,
                        potList=potList,
                        numSteps=200*scale_anneal_time,
                        finalTime=.4*scale_anneal_time,
                        printInterval=100)

    cool.run()

    protocol.initMinimize(dyn, printInterval=50)
    dyn.run()

    protocol.initMinimize(minc,
                        potList=potList,
                        dEPred=10)
    minc.run()


from simulationTools import StructureLoop, FinalParams
StructureLoop(numStructures=numberOfStructures,
              doWriteStructures=True,
              pdbTemplate=outFilename,
              structLoopAction=calcOneStructure,
              genViolationStats=True,
              averageTopFraction=0.5,
              averageSortPots=[potList['BOND'],potList['ANGL'],potList['IMPR'],noe],
              averageContext=FinalParams(rampedParams),
              averageCrossTerms=refRMSD,
              averageFilename="SCRIPT_ave.pdb",
              averagePotList=potList).run()
```

References

1 Furtig, B., Richter, C., Bermel, W. & Schwalbe, H. New NMR experiments for RNA nucleobase resonance assignment and chemical shift analysis of an RNA UUCG tetraloop. *J Biomol NMR* **28**, 69-79, doi:10.1023/B:JNMR.0000012863.63522.1f (2004).

2 Wolter, A. C. *et al.* NMR resonance assignments for the class II GTP binding RNA aptamer in complex with GTP. *Biomol NMR Assign* **10**, 101-105, doi:10.1007/s12104-015-9646-7 (2016).
